# Ossification of the Posterior Longitudinal Ligament Caused by X-linked Hypophosphatemia

**DOI:** 10.31662/jmaj.2023-0006

**Published:** 2023-05-10

**Authors:** Koichiro Yamamoto, Kosei Hasegawa, Takao Yasuhara, Fumio Otsuka

**Affiliations:** 1 Department of General Medicine, Okayama University Graduate School of Medicine, Dentistry and Pharmaceutical Sciences, Okayama, Japan; 2 Department of Pediatrics, Okayama University Hospital, Okayama, Japan; 3 Department of Neurological Surgery, Okayama University Graduate School of Medicine, Dentistry and Pharmaceutical Sciences, Okayama, Japan

**Keywords:** ectopic ossification, hereditary rickets, *PHEX*

A woman in her fifties with X-linked hypophosphatemia (XLH) presented deteriorating symptoms such as neck discomfort and numbness in the limbs. She was diagnosed with XLH with a novel heterozygous variant of the phosphate regulating endopeptidase X-linked gene (c.732 + 2delT) by us six months prior. Burosumab administration improved the hypophosphatemia; however, her symptoms gradually worsened. Two months ago, she accidentally fell. Subsequently, knee buckling during gait and stiffness in the trunk became prominent. Spine computed tomography revealed ossification of the posterior longitudinal ligament (OPLL) between C2 and T1 (Figure 1A). Furthermore, spine magnetic resonance imaging revealed spinal cord compression at C3/4 and C6-T1 (Figure 1B). Laminectomy was successfully performed from C2 to T1; consequently, neurological symptoms were ameliorated.

**Figure 1.**
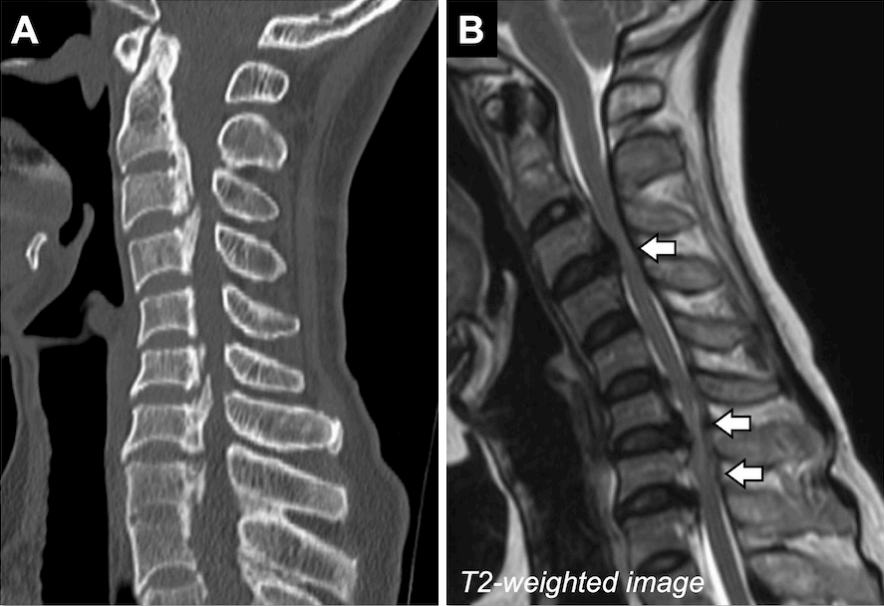
A) Spine computed tomography showed ossification of the posterior longitudinal ligament from C2 to T1. B) Magnetic resonance imaging of the cervical spine revealed intramedullary high signal intensity on T2-weighted images at the C3/4 and C6-T1 levels (arrows).

In this case, burosumab therapy improved the hypophosphatemia; however, symptoms worsened after the accidental fall, which OPLL is typically associated with. Adult XLH patients, specifically patients over the age of 40 years, have been reported to have a high OPLL prevalence ^(1)^. Inadequate mineralization of bone caused by hyperparathyroidism due to XLH may induce spinal ligament ossification ^(2)^. Thus, this case highlighted OPLL as an important complication, which may require surgery in symptomatic adult XLH patients. OPLL may be used for detecting undiagnosed hypophosphatemic osteomalacia.

## Article Information

### Conflicts of Interest

None

### Author Contributions

KY wrote the first draft and managed the submission process. KH and TY contributed to the clinical management of the patient and revised the manuscript. FO organized writing of the manuscript.

### Informed Consent

Written informed consent was obtained from the patient to publish this report.
